# Third Year MBBS - Gateway to Clinical Skills Learning

**DOI:** 10.31729/jnma.8347

**Published:** 2023-11-30

**Authors:** Sujata Ghimire, Suvam Lahera

**Affiliations:** 1 Nepalese Army Institute of Health Sciences, Sanobharyang, Kathmandu, Nepal

**Keywords:** *clinical skills*, *communication*, *empathy*

## Abstract

Practising medicine is all about art, skills and knowledge. According to the Tribhuvan University curriculum, the second phase, third year Bachelor of medicine and bachelor of surgery is the beginning of clinical skill learning. After studying integrated basic medical sciences for two years which is more like a conventional method of learning, the third year brings a new experience. It integrates theoretical knowledge in hospital settings by observing the patients, historytaking, examination, learning effective communication skills and empathy.

## INTRODUCTION

History taking, physical examination, communication and empathy are the basic ingredients of medical education.^1^ Third year is the gateway to clinical skill learning with the foundation of knowledge of pre-clinical subjects.^2^ Continuous lecture hours tend to get dull and tedious but the third year brings a pause to this. We are exposed to hospitals for patient observation, history taking, physical examination and communication with them for the first time in the third year. We are posted in paediatrics, obstetrics and gynaecology, surgery, and internal medicine for nine weeks each in two rotations. Since we are expected to perform in real-life situations, more than theoretical knowledge, clinical postings, regular interaction with patients and prior knowledge of basic science theories play a pivotal role. It brings confidence and provides us with skills that are integral to patient care. Emotions play a significant role in human interactions, yielding communicative intentions, modelling behaviour, promoting attachment, influencing information processing and determining choices so it is also an equally important part of medicine.^3^

## EXPERIENCE

After the first two years of medical school which focuses on basic medical science, the third year brings new opportunities and challenges at the same time. Communication with patients and the patient's party, making them convinced to give consent to know their health status was a tough task initially. What we studied about doctor-patient relationships during our preclinical years made real sense for gaining adequate history, examination, compliance, communication and empathetic feelings towards their suffering.

Our teachers always emphasised the fact that history taking is an art and a perfect history plays a major role in diagnosis so we are expected to at least learn the basics by the end of the third year. For that daily interaction with patients, knowing the basic format of history taking in different postings, a strong foundation of basic science and repeated practice is important. Prior knowledge about the pathophysiology, and clinical features of diseases and discussions with friends create a significant difference. Self-directed learning like this makes us conscious and more engaged while learning and also helps to explore in depth about any topic. Such practice should be incorporated from the 1st year itself to make it even more productive.^4^ These help us to integrate our knowledge of basic sciences with clinical concepts and problem-based learning. It helps to relieve the stress of the students on patient handling and provides realtime clinical reasoning abilities, communication skills, professional attitude and empathy.^5^ It has been found that early clinical exposure motivates the students in various ways, increasing their academic strength and improving clinical skills.^6^ From wards, outpatient department to neonatal intensive care unit, operation theatre and Emergency we observed, practised, and learnt the fundamental concepts, moral attitudes, communication skills and empathy.

During our obstetrics and gynaecology posting, we observed a case of a married female who had to undergo a total abdominal hysterectomy for cervical cancer. The patient and family members were counselled by an attending gynaecologist about the disease, its progression, treatment methods available and its consequences. For a young female who hadn't completed her family, the fact that she couldn't conceive and have a baby came out to be devastating and unacceptable at the same time but after the discussion on the fact that the benefit outweighs the risk she agreed for the surgery and it was successful as well. This provided us with powerful insights into the importance of counselling, understanding patients and their families' emotions, and offering concern and care is equally important as correct diagnosis and treatment.

Learning medicine is an intensely intellectual endeavour, demanding that you learn and understand an enormous body of information and that you constantly update that information as new knowledge becomes available but it is also an endeavour of the heart.^7^ We need to be prepared for innumerable ups and downs as there are some trying situations that we witness with patients and their families and what they are going through. We need to bring both the rational and empathetic aspects to bear when we are caring for patients. To handle the situations appropriately, we also need to think, act, apply, and learn to emotionally detach ourselves.

Sometimes, it is fun interacting with patients, learning from their experiences and a sense of gratitude towards their positive attitudes. Apart from all these we also got chance to observe modified radical mastectomy, emergency caesarean section, ruptured ectopic pregnancy, vaginal delivery, biopsy for endometriosis, neonatal jaundice, multiple myeloma, cannula insertion, cardiopulmonary resuscitation, performed vaccination in paediatrics population and so on. It helped us to establish the cognitive component of professional learning by providing significance to the basic science knowledge, expansion of knowledge and emphasising learning by doing.^8^

Exam patterns are entirely different from preclinical years which are more clinical, case-based questions, bedside examination, viva, voice, instruments and drugs. Not just in academics, the third year is a great opportunity to indulge ourselves in research activities, volunteering activities, health camps, personal development, explore career options and other extracurricular activities hence we can make much out of it. The medical field is constantly evolving, and patients' expectations are changing. If we learn the basics in the third year itself, it will be quite easy in the coming years to add on and update knowledge on our previous concepts and better understanding of diagnosis and management of the disease. This can help us in saving our time.

However, various factors should be considered in developing countries like ours in delivering an effective environment for making our learning experience better. The lack of clinical orientation, abrupt transition from preclinical to clinical years, ineffective feedback delivery system, lack of supervision and inadequate resources are the barriers to clinical learning. Reforms in the field of medical education in terms of integration of clinical curriculum, strengthening of the educational structure, and development of an effective feedback delivery system will help to reduce these barriers and improve the quality of clinical learning.

## WAY FORWARD

Lectures are equally important but reinforcing it with observation of patients and their disease condition helps us to learn more and gain confidence with a deeper understanding of the medical profession. Dedicating our efforts to learning basic skills and the art of history taking, examination and adequate knowledge would be rewarding in our medical career. We tend to get excited observing new cases every day, but it is equally important to empathise with their problem and maintain their confidentiality.
